# Exploring neurodevelopment through oscillatory and aperiodic EEG activity: methodological and clinical consideration

**DOI:** 10.3389/fnhum.2025.1641840

**Published:** 2025-09-02

**Authors:** Stefania Petri, Helene Vitali, Claudio Campus, Martina Riberto, Monica Gori

**Affiliations:** ^1^Italian Institute of Technology, U-VIP Unit for Visually Impaired People, Genoa, Italy; ^2^Department of Informatics, Bioengineering, Robotics, Systems Engineering (DIBRIS), University of Genoa, Genoa, Italy

**Keywords:** EEG spectral analysis, oscillatory, aperiodic, neurodevelopment, children

## Abstract

Understanding the neural dynamics that characterize early brain development is a central goal in neuroscience. Electroencephalography (EEG), is particularly well suited for studying the developing brain, given to its non-invasive nature and high temporal resolution. Spectral analysis of EEG signals reveals two key components: oscillatory activity associated with cognitive processes such as attention, memory, and perception; and aperiodic activity that reflects scale-free neural processes. While traditionally neglected, the aperiodic component has recently emerged as a crucial feature in understanding both typical and atypical brain functioning. This narrative review provides an overview of the most recent evidence regarding these EEG spectral features with a focus on their developmental trajectories and clinical significance. The review is structured into three main sections: (i) methodological considerations for analyzing oscillatory and aperiodic components of EEG spectrum; (ii) developmental changes in EEG spectral features during early childhood; (iii) alterations in spectral activity in children with developmental disorders. By highlighting recent findings and identifying gaps in the literature, this review aims to advance our understanding of how spectral EEG analysis can inform both basic and clinical neuroscience in early development. We emphasize the importance of considering both oscillatory and aperiodic components as a complementary feature of the spectral characteristic of the EEG signal contributing specifically to the characterization of brain functioning. In sum, this review offers updated and comprehensive support for researchers and clinicians working in developmental neuroscience serving both as a starting point for new studies and a bridge toward more effective EEG-based clinical tools.

## 1 Introduction

Identifying transdiagnostic biomarkers that underline various neurodevelopmental disorders represents one of the emerging challenges in clinical neuroscience. In line with dimensional frameworks (e.g., Research Domain Criteria, RDoC), the goal is to focus on underlying brain systems and behavioral functions, rather than relying solely on traditional diagnostic categories (Morris et al., 2022). Neurodevelopmental trajectories in healthy populations, whether and how they differ in children with atypical development of brain functions remain open questions. One of the reasons for this gap in literature is the technical challenges associated with recording brain activity in young populations. Among the recording techniques, EEG and magnetic resonance imaging (MRI) require a high degree of compliance from participants because of the large number of stimuli and extended duration of the experimental procedure to assure sufficient statistical power and reliable results. In addition, head movements can affect the quality of the signal recorded, thus requiring participants to assume a still position for a long time (Chugani, 2018; Gilmore et al., 2012; Holland et al., 2014). Compared to MRI, EEG offers a simpler setup and reduced costs, making it suitable for use in different contexts and adaptable to a wide range of experimental paradigms. Its high temporal resolution, and relative ease of application with infants and children make it especially valuable for measuring the dynamics of the developing brain (Fox et al., 2024; Grootjans et al., 2024).

A growing body of research focused on oscillatory and aperiodic components of EEG spectral analysis as promising biomarkers to investigate brain functioning from the earliest stages of life. Oscillatory components, namely the rhythmic brain activity typically represented by distinct frequency bands, have traditionally been the focus of EEG spectral analysis for understanding the brain functioning (Buzsáki and Draguhn, 2004; Im, 2018; Jensen et al., 2002). In contrast, the aperiodic component (Deodato and Melcher, 2024; Ossandón et al., 2023), often regarded as mere background noise, has only recently gained interest, as it provides complementary information about brain dynamics and neurodevelopmental trajectories. Unlike periodic components, which reflect oscillatory network activity by a specific time scale (as described by the relationship *f* = *1/T*), aperiodic components are scale-free, representing dynamic processes not limited to a particular frequency (Wen and Liu, 2016). Both components emerged through spectral decomposition of the EEG signal, which reflects the neural firings of large neuronal populations distributed across the cortex with high temporal resolution (Wright and Liley, 1996). Advancements in signal detection technologies and data analysis have also greatly improved the utility of EEG in pediatric populations, providing more information about brain function for a deeper understanding of the pathophysiology underlying various psychiatric and neurological disorders (Tremblay et al., 2019). In this regard, recent findings show that the analysis of EEG spectral characteristics in developmental ages has proven to be crucial for understanding the clinical manifestations of various developmental disorders (Adelhöfer et al., 2021; Cañigueral et al., 2022; Wang et al., 2017).

In this narrative review, we focus on the spectral characteristics of the EEG signal as promising biomarkers to investigate brain functioning from the earliest stages of life (Attar, 2023; Bell and Cuevas, 2012; Clements et al., 2021; Hervé et al., 2022; Waschke et al., 2021; Zhang et al., 2023) and how this might change in neurodevelopmental disorders. With this aim, we will describe the technical aspects related to both the oscillatory and aperiodic components of the EEG signal and discuss their functional meaning for understanding the brain function. Then, we will emphasize the potential of oscillatory and aperiodic components of EEG spectral analysis for the study of the developing brain from the 1st years of life. Finally, we will conclude by showing changes in spectral EEG activity in children with developmental disorders.

## 2 The methodological and functional aspects of the EEG spectrum

### 2.1 The decomposition of the EEG spectrum

EEG spectral analysis is the method typically used to decompose the EEG spectrum and quantify neural activity across different frequency bands (rather than across time points) to examine energy fluctuations and the underlying neural processes (Zhang et al., 2023). To characterize the frequency composition of EEG signals, mathematical transformations, such as the Fast Fourier Transform (FFT), have been employed. The FFT computes the Discrete Fourier Transform (DFT), which decomposes the time-domain signal into its constituent sinusoidal components. The resulting Fourier coefficients represent the amplitude (magnitude) and phase of oscillatory activity at each frequency, providing crucial information for understanding the spectral structure of the neural signal (Gao, 2016; Stoyanov et al., 2023). From the frequency domain, the Power Spectral Density (PSD) can be computed by squaring the magnitude of the Fourier coefficients obtained from the FFT. The PSD quantifies how the power of the EEG signal is distributed across different frequency bands of interest, providing a characterization of the signal's frequency composition. It can be expressed in terms of absolute or relative power. The former refers to the direct amount of spectral power within a given frequency band and provides a direct measure of the amplitude of band-filtered neural activity (Carrier et al., 2001; Govindan et al., 2017); the latter expresses the power in each frequency band as a proportion of the total spectral power (Markovska-Simoska and Pop-Jordanova, 2017). While both metrics are complementary, the decision to use one measure over the other depends on the specific research questions. An increase in absolute power in a frequency band often reflects a general rise in brain activity, such as heightened alertness or overall activation. Therefore, this measure is particularly valuable in longitudinal study (Soininen et al., 1991). In turn, an increase in relative power, even with stable total absolute power, typically signals a reorganization of brain activity where that specific frequency gains dominance. Thus, relative power is more sensitive to variations in spectral distribution and better suited for comparing individuals or conditions, especially when assessing developmental or state-related changes (Niemarkt et al., 2011; Yoon et al., 2021). This distinction is exemplified in alpha rhythm maturation: as we grow, relative alpha power increases, mirroring structural changes in cortico-cortical and thalamocortical connectivity, while the overall alpha oscillatory power decreases due to synaptic pruning processes (Cragg et al., 2011; McSweeney et al., 2023; Tröndle et al., 2022). Furthermore, the topographical mapping of the activity (i.e., magnitude or power values) could provide useful information about the functional interpretation of these metrics, since differences between specific bands and brain regions may reflect distinct neural mechanisms, sensorimotor or cognitive processes (Im, 2018; Schomer and Lopes Da Silva, 2017). However, the spatial resolution of such interpretations remains limited unless appropriate source reconstruction methods and validation procedures are applied (Mahjoory et al., 2017). Considering the potential of EEG spectral decomposition, below we focus first on the oscillatory component, those rhythmic patterns traditionally defined by canonical frequency bands, which have long been central to the study of brain function and development. This will serve as background to introduce the second and far less studied measure, the aperiodic component, that as complementary biomarkers underlying brain development and neurodevelopmental disorders.

### 2.2 Oscillatory spectrum components

The oscillatory component of the EEG spectrum refers to the rhythmic brain activity, typically represented by distinct frequency bands. Following traditional practices, the canonical frequency bands are typically defined as: Delta (0.5–4 Hz), Theta (4–8 Hz), Alpha (8–12 Hz), Beta (12–30 Hz), Gamma (30–60 Hz; Donoghue et al., 2020b). The power of these frequency bands has been shown to correspond to various brain functions, such as sensory processing, memory, attention, and motor control (Başar, 1999; Clements et al., 2021; Doppelmayr et al., 2002; Marosi et al., 2002; Ward, 2003). For example, it has been shown that Delta activity during wakefulness increased over frontal regions in cognitively demanding tasks, possibly reflecting a mechanism that inhibits external sensory input to sustain focused attention (Harmony, 2013). Delta activity has also been linked to autonomic regulation, motivational and emotional processing (Knyazev, 2007; Knyazev et al., 2009). Theta activity presents in two distinct patterns: a diffuse increase across the scalp (especially in fronto-central areas), typically linked to drowsiness and decreased cognitive efficiency, and frontal midline component, associated with sustained attention and mental effort, likely generated in the anterior cingulate cortex (Snipes et al., 2022; Tan et al., 2024). Posterior Alpha activity, typically observed over occipito-parietal regions during relaxed wakefulness, decreases with visual input and mental effort; lower Alpha (8–10 Hz) has been linked to attention, while upper Alpha (10–13 Hz) is more involved in semantic memory processing and stimulus-specific expectancy (Jensen et al., 2002; Morrow et al., 2023; Van Diepen et al., 2019). Although its specificity and reliability as a marker of mirror neuron system involvement remain debated, an alpha-like rhythm called mu rhythm (8–13 Hz), is recorded over sensorimotor areas and is suppressed during action execution or observation and has been related to motor control and mirror neuron system activity, with implications for learning and social cognition (Fox et al., 2016; Frenkel-Toledo et al., 2014). Beta activity shows fronto-central distribution and has been associated mainly with sensorimotor activity. However, some studies evidenced its role also in cognitive and emotional processes. It increases with attention, motor inhibition, static motor control, and is reduced during movement transitions (Baravalle et al., 2018; Brinkman et al., 2014). Finally, Gamma activity, though more difficult to detect with scalp EEG, has been linked to attention, sensory integration and large-scale neural synchrony, often nested within theta cycles and involving regions, such as te anterior cingulate cortex (Fries, 2015; Jensen et al., 2007).

Oscillatory activity has been often studied by comparing the power within a specific frequency band across groups or conditions, as it is thought to reflect distinct neural processes associated with different functional states (Başar, 1999; Brenner et al., 1986). However, some findings suggested that the functional relevance of neural oscillations extends beyond power alone. Indeed, oscillations contribute to neural computation by modulating the timing and excitability of neuronal populations. For instance, the phase of oscillation can influences when a neuron is more likely to fire, thereby enabling phase-dependent gating of sensory input. This dynamic modulation supports mechanisms such as input selection, attention, and the coordination for distributed neural assemblies through temporally precise synchronization (Buzsáki and Draguhn, 2004; Uhlhaas and Singer, 2010). These dynamic properties highlight that oscillatory activity is also an active mechanism for organizing different neuropsychological functions and related neural networks. The long-standing focus on oscillatory activity is due to its spectral prominence, appearing as distinct peaks in the power spectrum, and its consistent association with cognition and behavior (Başar, 1999; Marosi et al., 2002). Consequently, oscillations have shaped theoretical models of brain function and have served as important markers in both research and clinical context.

While oscillatory components have been the primary focus of EEG spectral analysis, recently there has been a growing interest in the aperiodic spectrum component of the EEG signal. Therefore, in the next section, we will focus on these measures of the EEG spectrum, which provides complementary information about brain dynamics that could not be detected with the oscillatory components alone.

### 2.3 Aperiodic spectrum component

The Aperiodic spectrum component refers to the broadband, “scale-free” component of the EEG power spectrum, often regarded as mere background noise, has traditionally received considerably less interest (Baranauskas et al., 2012; Voytek and Knight, 2015). Only recently, this perspective evolved, leading to growing attention to the aperiodic signal's functional relevance. Ostlund et al. demonstrated that the parametrization of the Power Spectral Density (PSD), which quantifies how the power of the EEG signal is distributed across different frequency bands of interest, enables the separation of its oscillatory and aperiodic elements, each capturing distinct aspects of neural dynamics (Ostlund et al., 2022). Moreover, an increasing number of studies have emphasized that this scale-free component may account for complementary aspects of brain activity not evidenced by the oscillatory activity alone (Brake et al., 2024; Demuru and Fraschini, 2020; Liu et al., 2023).

**Figure 1:**
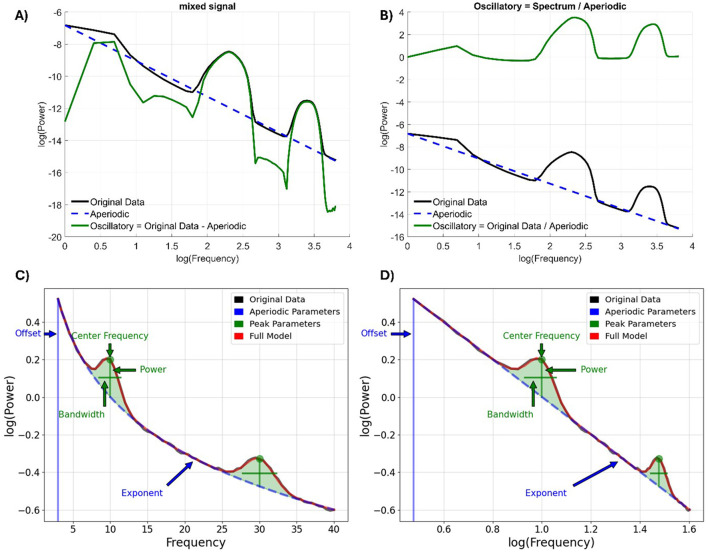
**(A)** Original implementation by Donoghue et al. (2020b) operates through the semilog-power (linear frequency, log-power) space and transformed back into linear-linear space. **(B)** defining an alternative expression for the oscillatory component as the quotient of the power spectrum and the aperiodic component. **(C)** Original data, aperiodic and oscillatory components and the full model (summing aperiodic and oscillatory components). The aperiodic component is characterized by the offset (i.e., intercept) and the exponent (i.e., slope) parameters. Instead, each oscillatory component is characterized by peak parameters, namely the center frequency of the peak, in units of frequency; the power of the peak, over the aperiodic component, in units of power; the bandwidth, or the width of the peak, in units of frequency. **(D)** the same representation in the log-log space. Figure adapted using code from the FOOOF Python toolbox (Donoghue et al., 2020b) and the FieldTrip toolbox (Oostenveld et al., 2011).

From a methodological point of view, different algorithms were designed for this purpose (Farrokhi and Daliri, 2022; Wen and Liu, 2016). Among the most widely used, *Fitting Oscillations and One-Over-F* (FOOOF, see Figure 1), renamed as *SpecParam* (Ostlund et al., 2022), models the power spectrum as a combination of Gaussian peaks, representing oscillatory activity superimposed on a 1/f-like background that captures the aperiodic structure (Donoghue et al., 2020b). This approach enables the estimation of two aperiodic parameters. The *slope* indicates how power decreases across frequencies on a log-log scale and is increasingly interpreted as an indirect marker of the excitation/inhibition (E/I) balance in neural populations. Specifically, a steeper slope suggests greater inhibitory activity, while a flatter slope is associated with increased excitatory modulation (Gao et al., 2017; Gyurkovics et al., 2022; Manning et al., 2009; Stanyard et al., 2024). On the other hand, the *offset* represents the overall vertical shift of the power spectrum and captures broadband changes in neural activity, often linked to aggregate neuronal firing rates and synaptic activity (Miller et al., 2009). Together, these parameters offer a complementary perspective to traditional oscillatory analyses, providing information into large-scale cortical dynamics from both neurophysiological and cognitive viewpoints.

Mathematically, this component follows a power-law relationship, expressed as P ∝ 1*/f* ^β^, with P representing Power (corresponding to the offset), *f* is frequency, and β is the power-law exponent (denoting to the slope; He, 2014). The latter describes how quickly the power decreases with frequency, visualized as the negative slope of the spectral plot when shown in log-log space. Unlike oscillatory activity, which is confined to narrow frequency bands, the aperiodic signal contributes to a continuous, scale-free component across the entire spectrum, shifting attention from discrete rhythms to broader spectral dynamics (Donoghue et al., 2020a).

It has been demonstrated that these aperiodic parameters are sensitive to cognitive processes and developmental changes, providing new relevant information to understand brain maturation mechanisms during early childhood (Ostlund et al., 2022). Specifically, aperiodic activity exhibits a gradual flattening of the power spectrum across development, reflecting shifts in cortical E/I balance and reductions in broadband neural activity, particularly from infancy through adolescence (Karalunas et al., 2022). Functionally, these parameters are associated with core cognitive processes, such as working memory and decision making, and contribute to perception, attention, and the regulation of cognitive effort (Lu et al., 2024; Thuwal et al., 2021). Taken together, these findings underscore the importance of incorporating the aperiodic component into EEG spectral analyses, as it provides a more comprehensive view of brain activity. This approach complements traditional oscillatory measures and offers additional perspectives on the functional and developmental properties of the brain throughout the lifespan.

The next section examines how both oscillatory and aperiodic components of the EEG spectrum contribute to our understanding of brain development, beginning with the role of oscillatory activity in early stages of life.

## 3 Understanding brain development through EEG spectral activity

### 3.1 Oscillatory spectrum component in the developing brain

The study of brain activity throughout development has been a major focus of scientists since the earliest observation of EEG rhythms in humans. This interest reflects the profound maturational changes that occur in the human brain from the prenatal period through adolescence (Gunnar and Nelson, 2013; Lemaître et al., 2021; Tamnes et al., 2010). During the 1^st^ year of life, the brain undergoes rapid and dynamic transformations, including the proliferation of synaptic connections, dendritic growth and the progressive myelination of white matter (Cao et al., 2017; Dubois et al., 2014). These processes follow a posterior-to anterior trajectory, with sensory and motor areas developing before higher-order associative cortices (Paus et al., 2001).

Alongside neuroanatomical changes, EEG spectral analysis has revealed corresponding changes in neural activity. The oscillatory components of the spectrum demonstrated how changes in brain rhythms reflect shifts in children's sensorimotor and cognitive abilities (Perone et al., 2018). During the first 7 months of life, the EEG spectral activity is predominantly characterized by low-frequency rhythms, such as delta (0.5–4 Hz) and theta (4–8 Hz; Lindsley, 1938; Schaworonkow and Voytek, 2021). Over time, higher frequency rhythms begin to emerge. Alpha activity, especially in posterior regions, becomes increasingly prominent (Niedermeyer, 1997). In this regard, a recent meta-analysis by Freschl et al. (2022) provided the developmental trajectory of peak alpha frequency (PAF), highlighting a steady increase from approximately 6 Hz (within the lower theta range) in early infancy to an asymptote of 10 Hz by 18 years of age, where it reaches adult-like levels (Freschl et al., 2022). As children grow through childhood and adolescence, slow-wave rhythms (delta and theta) decline in amplitude, while faster rhythms such as alpha, beta, and gamma increase (John et al., 1980). This maturation of brain rhythms reflects the central nervous system's development, driven by the increasing connectivity and efficiency of subcortical-cortical and cortico-cortical networks (Ünsal et al., 2024).

Accordingly, oscillatory activity becomes increasingly specialized and context-sensitive with development. Although, some EEG rhythms show functional specificity already in newborns, such as selective cortical responses to upright face-like patterns shortly after birth (Buiatti et al., 2019), an early posterior component in the typical alpha band correlated with visual activity (Campus et al., 2021), and a specific involvement of mu and beta activity during action preparation and sensorimotor coordination in infants (Liao et al., 2015; Van Elk et al., 2008; Vitali et al., 2024a), the functional role of the brain rhythms mature with age. As children grow, the neural oscillations become increasingly organized, with improved temporal coordination among neuronal population (Cannon et al., 2014). This maturation is accompanied by broader neurophysiological changes: for example, a reduction in absolute spectral power with age has been linked to cortical thinning, while the emergence of faster oscillatory frequencies likely reflects increased myelination and axonal growth (Feinberg and Campbell, 2010; Whitford et al., 2007). However, the evolving spectral dynamics of the brain not only mark anatomical maturation, but also support the emergence of more refined perceptual and cognitive functions across development (Bhavnani et al., 2021; Chikhi et al., 2022). For example, neonatal EEG spectral profiles have been shown to predict attentional performance at school age in preterm children (Cainelli et al., 2021), and early oscillatory patterns have been linked to developing social, emotional, and cognitive abilities (Saby and Marshall, 2012).

Although the oscillatory component of the EEG shows important results, some studies have pointed out that these conventional spectral power measures present some methodological limitations, particularly when applied to infant data. For example, neural oscillations in early development frequently occur in transient bursts, and averaging power over extended time windows can lead to an underestimation of their true amplitude (Cole and Voytek, 2019; Donoghue et al., 2022). Furthermore, fluctuations in oscillatory frequency may result in reduced measured power, despite the presence of robust neural activity. To overcome these limitations, it is recommended to separate oscillatory components from aperiodic activity using methods, such as parametrized spectral decomposition (e.g., *SpecParam*) to improve the accuracy and interpretability of EEG findings (Donoghue et al., 2022, 2020b), particularly during development.

### 3.2 Aperiodic spectrum component in the developing brain

The influence of the aperiodic 1/f component on the EEG spectral activity represents an additional factor which can systematically bias frequency estimates, depending on parameters such as slope steepness, oscillation amplitude, and analysis parameters (Samaha and Cohen, 2022). Over the past few years, some studies show that both the aperiodic exponent and offset tend to decrease with postnatal age, revealing changes in the excitation-inhibition balance across different stages of the development, including infancy, childhood, adolescence and adulthood (Clark et al., 2024; Schaworonkow and Voytek, 2021). When considering first stages of development, Schaworonkow and Voytek (2021) demonstrated that changes in the aperiodic signal within the power spectral density are already detectable early in life. Analyzing EEG data from infants aged 1 to 7 months, they observed a decreasing slope of the power spectrum in the occipital-parietal region as a function of age reflecting rapid cortical maturation and growing attentional engagement (Schaworonkow and Voytek, 2021). Hill et al. (2022) showed that the slope of the power spectrum decreases with age from 4 to 12 years of life in eyes-open EEG recordings, while the offset shows a decline with age in both eyes-open and eyes-closed conditions (Hill et al., 2022). Favaro et al. (2023) examined a large sample of children aged 2 to 17 years and reported that both aperiodic exponent and offset decrease with age during wakefulness. These age-related reductions, together with the observed shift of maximal aperiodic activity from posterior to anterior regions, may reflect progressive cortical specialization and functional reorganization occurring throughout childhood and adolescence (Favaro et al., 2023). Recently, the sensitivity of these parameters to prenatal factors, such as gestational age, has also been highlighted, with longer gestation associated with steeper spectral slopes in both neonates and toddlers. These results indicate that gestational duration may have significant and potentially lasting effects on brain activity, raising important questions about possible behavioral and cognitive consequences (Luotonen et al., 2025).

Providing a broader perspective, a systematic review from Stanyard et al. (2024) synthesized the major findings on the developmental trajectory of aperiodic EEG parameters from birth to young adulthood (0–26 years), offering new comprehension into the maturation of E/I balance during early life. Among the parameters analyzed, this review evidenced that age-related changes in these measures follow complex, non-linear patterns. Specifically, authors report a rapid decline in aperiodic exponent during infancy, suggesting a developmental increase in cortical excitation relative to inhibition, followed by more heterogeneous fluctuation across later developmental stages. Additionally, the spatial distribution of maximal aperiodic activity shifts from posterior to anterior regions with age, possibly reflecting regional maturation of large-scale neural networks (Stanyard et al., 2024). However, our understanding of aperiodic EEG parameters remains a topic of active debate, as mixed results were also found. For instance, Wilkinson et al. (2024) reported increases, rather than decreases, in both aperiodic slope and offset during the 1st year of life (i.e., between 2 and 8 months of age). These increases were interpreted considering rapid postnatal neurodevelopmental processes, such as synaptogenesis, neuronal proliferation, and the maturation of inhibitory circuitry (Wilkinson et al., 2024). Interestingly, this upward trend in aperiodic slope contradicts with previous reports of decreasing slope even within infancy, suggesting that early developmental dynamics may be more complex and non-linear than previously thought. Such inconsistencies may arise from methodological differences, including EEG acquisition conditions, frequency range used in spectral parameterization, and age sampling windows.

To conclude, despite the lack of standardized guidelines (Im, 2018), EEG spectral analysis continues to provide valuable insights, remaining an effective tool for studying brain maturation during development and identifying potential developmental disorders (Ostlund et al., 2022; Pani et al., 2022). Recent advancements have further strengthened the clinical utility of EEG: a large-scale study by Iyer et al. (2024) introduced a machine learning-based measure of functional brain age (FBA), trained on EEG recordings acquired during sleep from over 1,000 children aged 1 month to 18 years. The FBA achieved high accuracy in estimating chronological age and revealed maturational delays in children with developmental disability such as Trisomy 21 (Iyer et al., 2024). These findings highlight the potential of EEG as a research tool as well as a clinical application, capable of capturing age-related neural dynamics and altered developmental trajectories. Altogether, the growing attention to spectral EEG metrics across development opens a concrete path toward their application in the early monitoring and profiling of atypical neurodevelopment.

## 4 The clinical role of EEG spectral activity in young children with developmental disorders

### 4.1 Oscillatory spectrum component in children with developmental disorders

The EEG oscillatory activity can provide additional objective information in the clinical application, both about the brain's functioning in the presence of pathology and the effects of therapeutic treatments (Livint Popa et al., 2020). Considering that brain plasticity is at its peak during the early years of life (Remer et al., 2017), EEG stands out as an essential tool for monitoring brain development and supporting clinicians in prompt medical and rehabilitative interventions. Here, we explore the role of the EEG spectral biomarkers in children with neurodevelopmental disorders (NDDs), genetic neurological syndromes, and sensory impairments within the pediatric population. NDDs are a highly prevalent and heterogeneous group of chronic conditions that emerge in early childhood and affect various domains of functioning (Francés et al., 2022). According to the DSM-5, NNDs include intellectual disability (ID), attention-deficit/hyperactivity disorder (ADHD), autism spectrum disorder (ASD), communication disorder (CDs), specific learning disorder (SLD), and motor disorder (MDs; American Psychiatric Association, 2013). Given the variability in clinical manifestation and the lack of specific outcome measures, EEG has become a widely used method to study brain function in NDDs, with an increase in research over the last years.

#### 4.1.1 Oscillatory EEG pattern in ASD children

Over the past decade, some studies have investigated resting-state EEG spectral power in infants with a familial likelihood of ASD, aiming to identify early neural biomarkers that could be associated with later ASD outcomes. In this context, Levin et al. (2020) provided salient evidence on the high short-term test-retest reliability of EEG spectral features in both ASD and typically developing children. Their findings support the notion that specific aspects of the power spectrum, such as alpha peak amplitude and frequency, can serve as stable and biologically meaningful markers, an essential prerequisite for biomarker development (Levin et al., 2020).

Moreover, other works suggest that atypical oscillatory activity, mainly in frontal regions, may reflect early alterations in brain development associated with ASD (i.e., deviations from age-expected changes in power across canonical frequency bands, such as reduced frontal alpha and gamma power; Huberty et al., 2021; Levin et al., 2017; Tierney et al., 2012). Piazza et al. (2023) further support earlier findings by showing that infants with a familial likelihood of ASD exhibit reduced EEG power in low-frequency bands, specifically delta and theta, already at 6 months of age, with persistent alterations in the theta band at 12 months. These differences were also evident in infants who later received an ASD diagnosis, demonstrating early reduction in spectral power as a potential marker of both risk status and diagnostic outcome. In contrast, the same study identified a distinct EEG profile in infants at higher likelihood of developing language learning impairment, characterized by increased absolute power in high-frequency bands (high alpha, beta and gamma), particularly at 12 months (Piazza et al., 2023). Alterations across frequency bands and brain states were also found in both wakefulness and non-REM sleep stages in preschoolers with ASD. Particularly, children with ASD exhibited reduced absolute alpha power over the left central region of the brain during wakefulness and lower slow and fast theta power during N1 sleep across parietal and temporo-parietal regions, alongside increased beta power in the left central-parietal region (Vasista et al., 2025). Considering another spectral feature, peak alpha frequency (PAF) has also emerged as a promising developmental biomarker. Dickinson et al. (2018) found that ASD children tend to exhibit a lower PAF compared to their typically developing peers, and that higher PAF was positively associated with higher non-verbal cognitive abilities within the ASD group (Dickinson et al., 2018). Edgar et al. (2019) further emphasized that this spectral aspect is sensitive to developmental changes, showing that the association between PAF and cognitive functioning are modulated by age. Specifically, while high PAF in younger children with ASD may reflect atypical early brain maturation, in older children it appears to relate more directly to high non-verbal intelligent quotient. These findings suggest that PAF could offer valuable complementary information on the neurodevelopmental trajectories associated with ASD cognitive functioning (Edgar et al., 2019). More recently, a systematic review by Hankus et al. (2025) examined EEG abnormalities in children with ASD over the past decade, focusing on their relationship with developmental, behavioral, and clinical features. The review found a high prevalence of EEG spectral anomalies such as atypical oscillatory activity in theta, alpha and beta bands, but highlighted that their clinical relevance remains unclear. The inconsistency of findings was attributed to limited data, lack of meta-analyses, and the heterogeneity of ASD clinical manifestations. Given the unclear clinical significance of EEG alterations in ASD, current evidence does not support routine EEG screening in all children; its use should be limited to cases with clinical suspicion of epilepsy (Hankus et al., 2025).

#### 4.1.2 Oscillatory brain activity in ADHD

Spectral EEG analyses in ADHD children have consistently revealed atypical patterns of oscillatory activity, particularly in the alpha, beta, and theta frequency bands. The spectral characteristics appear sensitive to individual differences in symptoms dimension, such as inattention and hyperactivity/impulsivity (Slater et al., 2022). Moreover, compared to neurotypical peers, ADHD children showed significantly weaker alpha power decreases, as well as attenuated increases in theta and beta power during cognitive tasks. These patterns were observed across multiple studies and task paradigms, reflecting a broad alteration of neural oscillatory mechanisms involved in attentional allocation and executive functioning (Michelini et al., 2022). Another oscillatory marker that has received particular attention in ADHD research is the theta-beta ratio (TBR). A meta-analysis by Arns et al. (2013) point out increased TBR in children and adolescence with ADHD as a possible diagnostic biomarker, despite substantial heterogeneity across studies and a reduction in group differences with age. This might suggest that while TBR may not be reliable for ADHD diagnosis, it holds a potential prognostic value for a specific subgroup of individuals with ADHD (Arns et al., 2013). In line with this, a prospective study by Begum-Ali et al. (2022) indicated that alterations in TBR can already be observed in infancy. Specifically, 10-month-old infants with a family history of ADHD exhibited lower TBR than those without genetic predisposition, and this early TBR profile was associated with temperamental traits linked to ADHD at 2 years, such as surgency, impulsivity and sociability. Notably, no such TBR alteration was observed in infants with an elevated likelihood of developing ASD, supporting a degree of specificity for ADHD. These evidences indicate the developmental sensitivity and potential predictive utility of TBR in early detection of ADHD-related neurodevelopmental trajectories (Begum-Ali et al., 2022).

#### 4.1.3 EEG markers in genetic syndrome during development

EEG studies in children with genetic disorders—often marked by cognitive delays, autistic features, and seizures—have shed light on underlying brain activity. Among these conditions, Down syndrome (DS) is the most common genetic condition associated with intellectual disability. It has been shown that DS preschool children differ from their age and developmental-matched peers in several oscillatory patterns, showing increased theta power, reduced alpha power and peak amplitude, and a lower prevalence of low-beta oscillations (Geiger et al., 2024). Other genetic conditions, such as Angelman and Rett syndromes, are associated with elevated delta power, which appears to correlate with disease severity, while Fragile X syndrome is characterized by increased in gamma-band and decreased alpha-band activities (Goodspeed et al., 2023). Additionally, in girls with Rett syndrome, a consistent increase in delta power has been observed, reflecting an alteration of the neural organization. The degree of EEG slowing in this population was also negatively correlated with cognitive performance, predicting more severe clinical phenotypes (Roche et al., 2019). In sum, EEG spectral analysis in genetic syndromes has proven to be a promising tool for identifying brain functioning characteristics that correlate with disease and functional impairment.

#### 4.1.4 Sensory processing and EEG oscillatory activity in visually impaired children

Research on EEG oscillatory dynamics has also highlighted how sensory experience can shape the developmental trajectories of brain activity. In this context, studies on children with visual impairments have shown how early visual deprivation impact the development of neural oscillatory activity, revealing peculiar EEG spectral patterns. For instance, Red'ka et al. (2014) compared children with congenital visual impairments to sighted peers aged 8–12 and reported globally reduced absolute spectral power across all frequency bands, particularly in the alpha range, alongside increased relative delta activity (Red'ka et al., 2014). Supporting these findings, more recent evidence suggests that visual experience plays a critical role in the maturation of alpha activity. While blind infants may initially exhibit posterior alpha oscillations, these are weaker and slower to develop compared to those in sighted peers, with marked differences emerging between the ages of 3 and 6 years. Moreover, blind children showed that reduced alpha activity is associated with an increased likelihood of motor impairments starting from the age of three. This highlights the critical role of sensory experience in shaping neural mechanisms related to action/perception and brain plasticity during early development (Campus et al., 2021). These results from awake EEG are complemented by a recent sleep-based EEG study, which demonstrated the impact of early visual deprivation on brain development. In blind and severely visually impaired children aged 5 months to 6 years, EEG during sleep revealed atypical maturation of fast sleep spindles in central regions, characterized by reduced high-sigma and high-beta activity. These alterations were also associated with perceptual and motor difficulties, indicating that spindle dynamics may serve as early biomarkers of atypical sensorimotor development (Vitali et al., 2024b). The findings on oscillatory activity in children with developmental disorders are summarized in Table 1.

**Table 1:** Summary table illustrating recent evidence on EEG spectral activity in developmental disorders.

	**Developmental disorder**	**Oscillatory activity**	**Non-oscillatory activity**
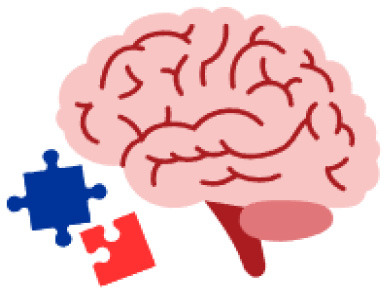	ASD	↓ frontal alpha and gamma power reflect early atypical development (Huberty et al., 2021; Levin et al., 2017; Tierney et al., 2012) ↓ low-frequency power at 6–12 m predictive of later ASD diagnosis (Piazza et al., 2023) ↓ alpha (wake), ↓ theta + ↑ beta (N1 sleep; Vasista et al., 2025) ↓ peak alpha frequency (PAF) in ASD children; ↑ PAF linked to ↑ non-verbal cognition in ASD (Dickinson et al., 2018; Edgar et al., 2019)	Steeper spectral slope in high-risk infants (Shuffrey et al., 2022)
	ADHD	attenuated task-related ↑ theta/beta and ↓ alpha power (Michelini et al., 2022) Atypical alpha, beta and theta activity associated with ADHD symptoms (Slater et al., 2022) ↑ theta/beta ratio (TBR) in children/adolescents with ADHD (Arns et al., 2013) ↓ TBR at 10 months in infants with familial ADHD risk; predicts ADHD-relevant traits at 2 y (Begum-Ali et al., 2022)	Steeper spectral slope negatively correlated with cognitive performances (Dakwar-Kawar et al., 2024) ↑ Offset, steeper slope in medication-naïve children (Robertson et al., 2019) Conflicting findings: flatter spectral slope (Arnett et al., 2024)
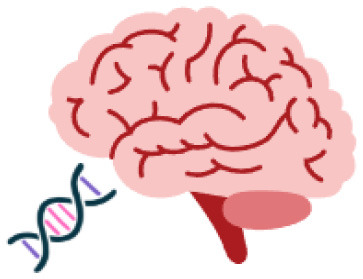	Rett syndrom	↑ delta power, reflects severity (Goodspeed et al., 2023)	Steeper spectral slope (Roche et al., 2019; Wilkinson and Nelson, 2021)
	Angelman syndrom	↑ delta power, reflects severity (Goodspeed et al., 2023)	Missing
	Fragile X syndrom	↑ gamma ↓ alpha (Goodspeed et al., 2023)	Steeper spectral slope (Roche et al., 2019; Wilkinson and Nelson, 2021)
	Down syndrom	↑ theta, ↓ alpha + beta (Geiger et al., 2024)	Flatter spectral slope (Geiger et al., 2024)
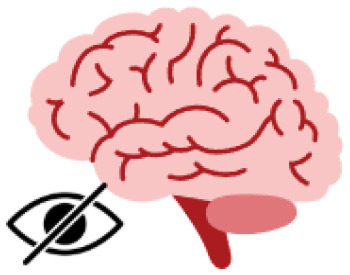	Visual impairment	↓ power across all bands; ↑ delta, ↓ alpha (Red'ka et al., 2014) ↓ alpha power, delayed alpha development, involving first alpha 1 and then alpha 2 (Campus et al., 2021) ↓ fast spindles (high sigma/beta) during sleep (Vitali et al., 2024b)	Missing

The green column presents findings related to the oscillatory component of the EEG signal, while the light blue column summarizes findings on the aperiodic (non-oscillatory) component of the EEG spectrum. Conditions are grouped into three categories based on the icons used in the first column: neurodevelopmental disorders (includes ASD and ADHD); genetic syndromes (includes Rett syndrome, Angelman syndrome, Fragile X syndrome, and Down syndrome); visual impairments.

Upward and downward arrows indicate increases or decreases in power or slope, respectively. Full references are provided for each finding.

### 4.2. Approaching developmental disorders through the aperiodic EEG spectrum component

In the previous section, we reviewed the major findings on EEG oscillatory activity and its features in developmental neuropsychiatric disorders. The existing literature on this topic is quite extensive, with most EEG spectral analysis studies focusing primary on the oscillatory component of the spectrum. Below, we focus on the aperiodic component, which has recently emerged as an informative biomarker of neurodevelopmental processes. Examining this component separately allows for the identification of neurophysiological features in developmental disorders that may be neglected by analyses focused solely on oscillatory activity.

#### 4.2.1 Aperiodic EEG activity in neurodevelopmental disorders and genetic syndromes

Most of the studies about the aperiodic component of EEG activity in clinical pediatric populations focused on children with neurodevelopmental disorders, particularly ASD and ADHD (Pani et al., 2022). Levin et al. (2020) demonstrated that resting EEG spectral features could serve as a stable biomarker of cortical activity in children with ASD, suggesting their diagnostic potential (Levin et al., 2020). To date, only a few studies have specifically examined aperiodic EEG activity in children with ASD. Although much of this work is still preliminary, the available evidence describes a relationship between aperiodic EEG characteristics and clinical manifestation of the pathological condition. One of the most recent and compelling findings comes from a study on preterm infants, which showed that the spectral slope of the aperiodic EEG activity was associated with increased autism risk at 3 years of age, while oscillatory features showed no such link (Shuffrey et al., 2022). The association between the spectral slope and later ASD risk aligns with findings from studies with genetic neurodevelopmental syndromes linked to ASD, such as Rett syndrome and Fragile X syndrome, where a steeper PSD slope has been observed (Roche et al., 2019; Wilkinson and Nelson, 2021). As regards ADHD disorder, Robertson et al. (2019) found that medication-naïve children showed altered EEG aperiodic parameters, characterized by steeper spectral slopes and greater offset, compared to typically developing controls and stimulant-treated ADHD children. Furthermore, these alterations in aperiodic features positively correlated with the theta/beta ratio, although the theta/beta ratio itself did not significantly differ between groups. These findings suggest that EEG aperiodic parameters could represent sensitive markers of neurophysiological alterations in ADHD, which may be modulated by stimulant medication (Robertson et al., 2019). In a more recent study, researchers further explored the role of aperiodic activity in ADHD by applying a spectral parametrization approach to resting-state EEG in children aged 6 to 12 years. They found that ADHD children exhibited significantly higher aperiodic exponent, interpreted as an indicator of reduced cortical excitation-inhibition balance, compared to typically developing peers. Moreover, the aperiodic exponent was negatively correlated with processing speed across the sample, suggesting its potential relevance as a neurophysiological marker of cognitive performance beyond oscillatory measures (Dakwar-Kawar et al., 2024). Interestingly, contrasting findings emerged from a large cross-sectional study involving children aged 2 to 14 years, which reported a decreased aperiodic exponent in the ADHD group compared to controls, suggesting a higher excitation-inhibition ratio, opposite to previous studies. This demonstrates the complexity of interpreting aperiodic activity in developmental populations and points to potential age-related factors influencing these measures (Arnett et al., 2024).

Emerging evidence also comes from the previously mentioned study on children with Down Syndrome, which additionally reported a flatter spectral slope compared to both age- and developmentally matched controls, particularly in frontal, central and posterior regions (Geiger et al., 2024). These findings support the idea of a cortical excitation increase, reinforcing the emerging role of the aperiodic exponent as an indirect marker of E/I balance across neurodevelopmental conditions.

#### 4.2.2 Non-oscillatory EEG patterns in visually impaired children

Finally, in the context of visual impairments during development, no studies to date have specifically investigated the aperiodic EEG spectrum component in early childhood. This clearly reflects how the inclusion of the non-oscillatory EEG activity in neurodevelopmental research is a very recent advancement. Further investigations are therefore needed to validate current findings and to better define how these parameters can be effectively studied and interpreted in clinical pediatric populations. In this context, our ongoing research within the ERC MySpace project (Grant Agreement No. 948349) is exploring both oscillatory and aperiodic components of the EEG spectrum in visually impaired infants and children, specifically to assess how altered visual input during the first stages of life can shape neurodevelopmental trajectories. The findings on aperiodic EEG component in children with developmental disorders are summarized in Table 1.

## 5 Conclusion

In this narrative review, we point at both oscillatory and aperiodic components of the EEG spectrum as promising biomarkers to investigate brain functioning from the earliest stages of life. This is in line with recent studies showing that both components could provide complementary information about brain development, and thus essential for understanding atypical neurodevelopmental trajectories (e.g., ASD, ADHD, genetic syndromes, congenital blindness) adopting dimensional frameworks. In particular, the oscillatory component reflects the organization of the neural networks, which communicate through traveling waves in a specific frequency band; the aperiodic component, previously ignored, recently gained attention as a marker of cortical E/I balance across the broadband neural activity.

Recent findings show that aperiodic parameters, such as the spectral exponent and offset, undergo significant changes across infancy and childhood in an experience-dependent fashion, shaped by factors like gestational age and sensory experience. Interestingly, in atypical developmental conditions such as ASD, ADHD and genetic syndromes, specific aperiodic spectral patterns have been associated with distinct cognitive and behavioral profiles, offering meaningful association with clinical outcomes.

While these advances highlight the clinical relevance of aperiodic activity consideration, research on children with visual impairments has predominantly focused on oscillatory features. Early sensory deprivation in this population has been linked to modifications in oscillatory patterns and sleep spindle-related EEG features, underscoring the influence of early sensory experience on brain development. Yet, the role of the aperiodic component in children with visual impairment remains largely unexplored, despite its potential to reveal changes in cortical dynamics not captured by oscillatory analysis alone. Thus, expanding the current body of research on the clinical application of EEG spectral analysis in early childhood is crucial to address several open questions, particularly in children with visual impairments, and for more inclusive and patient-tailored therapeutic interventions.
